# Determinants of Malaria Protective Immunity in Mice Immunized with Live Sporozoites during Trimethoprim–Sulfamethoxazole Prophylaxis

**DOI:** 10.4269/ajtmh.20-0749

**Published:** 2020-12-21

**Authors:** Charlotte V. Hobbs, Tejram Sahu, Jillian Neal, Solomon Conteh, Tatiana Voza, William Borkowsky, Jean Langhorne, Patrick E. Duffy

**Affiliations:** 1 Division of Infectious Diseases, Department of Pediatrics, Batson Children’s Hospital, University of Mississippi Medical Center, Jackson, Mississippi;; 2 Department of Microbiology, University of Mississippi Medical Center, Jackson, Mississippi;; 3 Laboratory of Malaria Immunology and Vaccinology, National Institute of Allergy and Infectious Diseases, National Institutes of Health, Bethesda, Maryland;; 4 W. Harry Feinstone Department of Molecular Microbiology and Immunology, Malaria Research Institute, Johns Hopkins University Bloomberg School of Public Health, Baltimore, Maryland;; 5 Biological Sciences Department, New York City College of Technology, CUNY, New York, New York;; 6 Division of Infectious Disease and Immunology, Department of Pediatrics, New York University School of Medicine, New York, New York;; 7 The Francis Crick Institute, London, United Kingdom

## Abstract

HIV and malaria geographically overlap. Trimethoprim–sulfamethoxazole (TMP-SMX) is a drug widely used in HIV-exposed uninfected and infected children in malaria-endemic areas, and is known to have antimalarial effects. Further study in terms of antimalarial impact and effect on development of malaria-specific immunity is therefore essential. Using rodent malaria models, we previously showed that repeated *Plasmodium* exposure during TMP-SMX administration, or chemoprophylaxis vaccination (CVac), induces CD8 T-cell–dependent preerythrocytic immunity. However, humoral immune responses have been shown to be important in models of preerythrocytic immunity. Herein, we demonstrate that antibody-mediated responses contribute to protective immunity induced by CVac immune sera using TMP-SMX in models of homologous, but not heterologous, parasite species. Clinical studies must account for potential anti-*Plasmodium* antibody induced during TMP-SMX prophylaxis.

## INTRODUCTION

HIV and malaria are highly prevalent together in many regions worldwide, and many HIV-exposed or infected children live in malaria-endemic areas. Trimethoprim–sulfamethoxazole (TMP-SMX) is a drug widely used to reduce opportunistic infection incidence in HIV-exposed and infected children, and, in parallel, this drug has antimalarial effect as an antifolate.^1^ However, despite its widespread use and antimalarial effects, its impact on malaria infection and the development of malaria-specific immunity in the context of widespread antifolate resistance, and in HIV-exposed uninfected and infected children, requires further study. Indeed, clinical studies have only recently begun to look at the impact of TMP-SMX prophylaxis duration on malaria as a reflection on immunity.^2,3^

Malaria infection with *Plasmodium* in the mammalian host begins with the female anopheline mosquito inoculating sporozoites (SPZs) during a blood meal. Sporozoites then travel to the liver and infect hepatocytes, where they develop into liver-stage or exoerythrocytic forms (EEFs). Exoerythrocytic forms produces tens of thousands of merozoites that then invade erythrocytes to initiate the blood stage. Naturally acquired immunity to malaria only develops after repeated malaria episodes and does not prevent reinfection. However, sterilizing anti-infection immunity can be induced with attenuated SPZs that arrest during liver-stage development.^4^ Anti-infection immunity is also observed in mice and humans that have been infected with SPZs and simultaneously given drugs that kill liver- or blood-stage parasites,^5,6^ and this process is termed chemoprophylaxis vaccination (CVac). Taken together, these data indicate that exposure to preerythrocytic parasites can induce highly effective protective immunity. Indeed, in mice, we have shown that TMP-SMX at prophylactic doses can arrest liver-stage development of *Plasmodium*, and that these regimens during repeated malaria exposure, termed CVac/TMP-SMX, induce protective immunity targeting preerythrocytic-stage parasites.^7^ Such immunity requires CD8 T cells, whereas the contribution of antibody remains incompletely investigated.^7^ Herein, we set out to determine the contribution of antibody to CVac/TMP-SMX–induced protective immunity using serum from mice in our established model of CVac with *Plasmodium yoelii*–infected mice, receiving TMP-SMX at levels which approximate what is achieved in children on standard TMP-SMX prophylaxis dosing.^7,8^

## METHODS

### Mice.

Female BALB/c mice, aged 4–6 weeks and weighing 20–25 g (Taconic or NIH), were used in experiments with approval from the NIAID/NIH Institutional Animal Care and Use Committee. Mice were age- and sex-matched for all experiments.

### Parasites and mosquitoes.

*Anopheles stephensi* mosquitoes were fed on mice infected with *P. yoelii* (17XNL) or *Plasmodium berghei* (ANKA), and SPZs were harvested from salivary glands on days 14–18 (*P. yoelii*) or days 21–26 (*P. berghei*) after mosquito dissection. All experiments were performed with *P. yoelii* 17XNL and *P. berghei* ANKA, except immunofluorescence assays (IFA) in which transgenic parasite lines expressing green fluorescent protein (GFP) (*P. yoelii* GFP [17XNL] and *P. berghei* GFP [ANKA]) were used.^9,10^ All mosquitoes had been fed on infected mice, and parasites had not undergone serial blood passages as this has been demonstrated to modulate infection virulence.^11^

### Trimethoprim–sulfamethoxazole regimen, SPZ inoculation, and efficacy assessment.

Trimethoprim–sulfamethoxazole was used in its commercially available generic suspension form and in a regimen that inhibits development of liver-stage parasites in vitro and prevents patent parasitemia in mice during CVac/TMP-SMX immunization.^7^ Our model of CVac, described in the following text, has been extensively explored immunologically and pharmacokinetically with *P. yoelii* and the immunization regimen used.^7,8^ Of note, *P. berghei* was not used for reinfection regimens that use TMP-SMX as some strains of *P. berghei* possess antifolate resistance.^1^

In brief, mice were injected three times with 10,000 *P. yoelii* 17XNL SPZs at 14- to 21-day intervals. Starting the same day as SPZ inoculation, immunized and control animals were given 60 μL of TMP-SMX by oral gavage at 18, 24, and 36 hours after SPZ inoculation. During the immunization regimen, the absence of parasitemia in immunized mice was confirmed by examination of Giemsa-stained thin blood smears from the tail blood every other day (up to day 15). Mice that received CVac/TMP-SMX were rested for a minimum of 4–6 weeks after the last SPZ and drug administration before serum collection.^7^ Immune sera obtained from *P. yoelii* CVac/TMP-SMX–immunized mice will be referred herein to as CVac sera.

Control sera were collected from malaria-naive mice that received no other intervention (“naive”) or received drug only but no SPZ inoculations (“drug control [DC]”). Separately, malaria-naive infectivity control mice received SPZs (in phosphate buffered saline [PBS] only) during each study to confirm parasite infectivity.

### In vitro and in vivo SPZ neutralization assays with CVac sera.

In vitro sporozoite neutralization assays (SNAs), which use serum to assess effect of the serum antibodies on parasite infectivity, were performed as previously described.^12^ In brief, 20,000 *P. berghei* or *P. yoelii* SPZs were preincubated on ice (*P. berghei*) or at room temperature (*P. yoelii*) for 40–45 minutes in sera pooled from groups of 5–10 CVac/TMP-SMX–immunized mice,^7^ or from DC or naive mice. Sporozoites were then added to confluent HepG2:CD81 cells (a gift of Dr. Eric Rubinstein, Inserm U935, Villejuif, France). Sporozoites prepared in 10–15 μL of dissection medium with 6 μL of sera were then incubated in a total volume of 30 μL (1:5 dilution) in each well in an 8-well chamber slide (Lab-Tek, Thermofisher Scientific, Waltham, MA)) with cultured cells, and then were incubated at 37°C for 60–72 hours with change of culture media (Dulbecco’s Modified Eagle Medium [DMEM] + 10% fetal bovine serum [FBS] + 100 U/mL penicillin–streptomycin) every 24 hours. Assays were run in quadruplicate wells per condition. At 60–72 hours, cells were trypsinized (trypsin–ethylenediaminetetraacetic acid [EDTA]) and washed twice with RNAse-free PBS, and total RNA was extracted, and parasite burden was estimated by qPCR. The total RNA was isolated using TRI reagent (Molecular Research Center, Cincinnati, OH), in accordance with the manufacturer’s instructions. Reverse transcription was performed using 1 µg of total RNA and random hexamers. Real-time PCR was performed using primers that recognize *P. yoelii*– or *P. berghei*–specific sequences within the 18S rRNA and the amplified product quantified using the QuantiTect SYBR Green PCR Kit (Qiagen, Hilden, Germany).^13^ Dilutions of 10-fold of a plasmid construct containing the *P. yoelii* and *P. berghei* 18S rRNA gene were used to create a standard curve. For PCR, experiments were run in triplicate, and three to four independent experiments were run.

For in vivo experiments, SNAs were performed with the incubation steps as earlier except that 40,000 SPZs in a 100-μL volume of PBS were preincubated in test sera at a final dilution of 1:5 for 45 minutes at room temperature.^14^ This was then diluted further in PBS to obtain a final concentration of 40,000 SPZs/mL. Naive mice were challenged with 100 μL of this solution, corresponding to 4,000 SPZs.^14^ Blood-stage patency was monitored for up to 15 days post-challenge. Two experiments with 5–10 mice per condition were run for each species of parasite.

### Immunofluorescence of SPZs and EEFs.

Immunofluorescence assays were performed on salivary gland SPZs as described elsewhere.^15^ Transgenic parasite lines expressing GFP (*P. yoelii* GFP [17XNL] and *P. berghei* GFP [ANKA]) were used for IFA experiments. In brief, 5 × 10^4^ SPZs in 200 μL obtained from mosquito salivary glands in dissection media were preincubated for 45 minutes in a polylysine-coated eight-well chamber slide (Lab-Tek) coated with confluent HepG2:CD81 cells. Sporozoites were then fixed with PBS-4% PFA for 10 minutes at room temperature. These SPZs were then used for immunostaining with or without permeabilization with 0.1% Triton X100 in PBS for 5 minutes at room temperature for SPZs, washed twice with PBS, and then blocked with PBS 3% bovine serum albumin (BSA) for 30 minutes at room temperature. These SPZs were then incubated with sera (1:400 in PBS-3% BSA) overnight at 8°C. After washing four times with PBS, SPZs were then incubated in RT with AlexaFluor 594-conjugated anti-mouse IgG (Thermofisher Scientific) for 2 hours at a dilution of 1:1,000 in PBS-3% BSA. Slides were then washed three times with PBS to remove unbound antibodies and finally with PBS with DAPI (Sigma-Aldrich, St. Louis, MO) 300 nM. Fluorescent images were acquired with confocal microscope (Leica SP5, Buffalo Grove, IL).

The immunofluorescence images of EEFs were acquired, as previously reported.^14^ In brief, 50,000 *P. yoelii*/*P. berghei* GFP SPZs/well were added onto confluent HepG2:CD81/HepG2 cells in eight-well chamber slides (Lab-Tek). After centrifugation for 5 minutes at 310 g at 20°C, slides were incubated at 37°C with culture medium as described earlier and changed twice daily postinfection. Developing EEFs were fixed at 48 hours, blocked with 3% BSA in PBS (with or without permeabilization with 0.1% Triton X-100 in PBS for 10 minutes), and stained with mouse sera as described earlier for SPZ IFA. Exoerythrocytic forms were labeled with chicken anti-EXP1 antibody to identify infected hepatocytes. Images were acquired using confocal microscope (Leica SP5).

### Statistical analysis.

Mann–Whitney tests were performed on combined experiments for in vitro assays. Survival curves were generated, and the log-rank (Mantel–Cox) test was used to compare groups. Data were normalized to naive controls across experiments and compared with DC for all statistical analyses. Statistical analyses were performed using GraphPad Prism software (version 7, San Diego, CA).

## RESULTS

### Chemoprophylaxis vaccination/Trimethoprim–sulfamethoxazole sera preincubation with *P. yoelii* SPZs, but not *P. berghei* SPZs, reduces liver-stage parasite burden in vitro.

**Figure 1.**
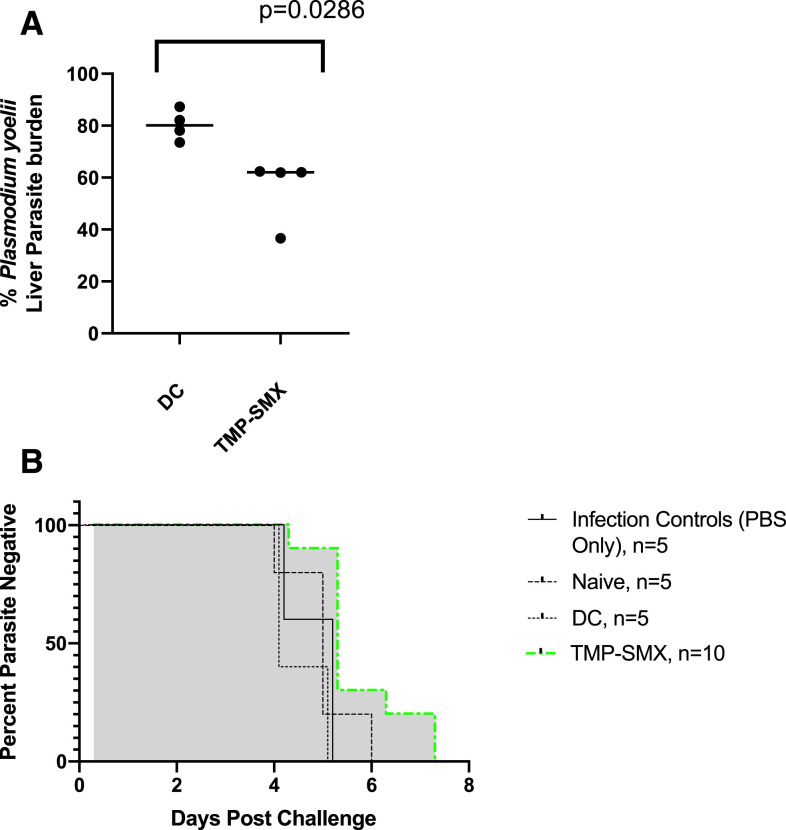
Antibody response generated by TMP-SMX-CVac are protective against homologous sporozoite infection. (**A**) Chemoprophylaxis vaccination (CVac) sera incubation with *Plasmodium yoelii sporozoites* (SPZs) results in reduced liver-stage parasite burden as measured by qPCR after in vitro infection. Shown is liver stage burden after in vitro sporozoite neutralization assay (SNA), with experiments conducted in quadruplicate, normalized to parasite burden estimated in SNA using naive mouse serum. DC = drug Control; TMP-SMX = trimethoprim-sulfamethoxazole-CVac immunized. *P* < 0.03 determined by Mann-Whitney test. Sporozoite neutralization assays (SNAs) demonstrated that incubation of *P. yoelii* SPZs with CVac sera resulted in significantly reduced liver-stage parasite burden in vitro, with reduction from median of 80% to median of 62% (A, P = 0.03, Mann–Whitney test). However, incubation of *P. berghei* SPZs with CVac sera did not reduce liver-stage parasite burden (data not shown). (**B**) CVac sera preincubation with *P. yoelii* SPZ delays but does not prevent patency. Shown herein are Kaplan-Meier plots representing data from in vivo SNAs, representative of two experiments with 5–10 mice per condition for each species of parasite. Preincubation of *P. yoelii* SPZs with CVac sera resulted in a significant delay in *P. yoelii* patency (day of detection of parasites in blood) compared with DC sera preincubation (*P* = 0.031, log-rank/Mantel–Cox test). By contrast, patency of heterologous *P. berghei* parasites was not significantly delayed (data not shown). Preincubation with sera from naive or DC mice with either parasite species did not affect mean days to patency compared with infection only controls (**B** for *P. yoelii*, data not shown for *P. berghei*). This figure appears in color at www.ajtmh.org.

In SNAs, *P. yoelii* SPZs incubated and overlaid on HepG2:CD81 cells with CVac sera yielded significantly reduced liver-stage parasite burden in vitro (Figure 1A, *P* < 0.03). However, similar incubation of *P. berghei* SPZs with CVac sera failed to reduce liver-stage parasite burden (data not shown).

### Chemoprophylaxis vaccination sera preincubation with *P. yoelii* but not *P. berghei* SPZs increases time to patency in vivo.

Preincubation of *P. yoelii* SPZs with CVac sera resulted in a significant delay in *P. yoelii* patency (day of detection of parasites in blood) compared with DC sera preincubation (Figure 1B, *P* = 0.03). By contrast, patency of heterologous *P. berghei* parasites was not significantly delayed (data not shown). Preincubation with sera from naive or DC mice with either parasite species did not affect mean days to patency compared with infectivity controls (Figure 1B for *P. yoelii*, data not shown for *P. berghei*).

### Chemoprophylaxis vaccination sera reacted with *P. yoelii* SPZs and EEFs but only EEFs of *P. berghei* by IFA.

**Figure 2.**
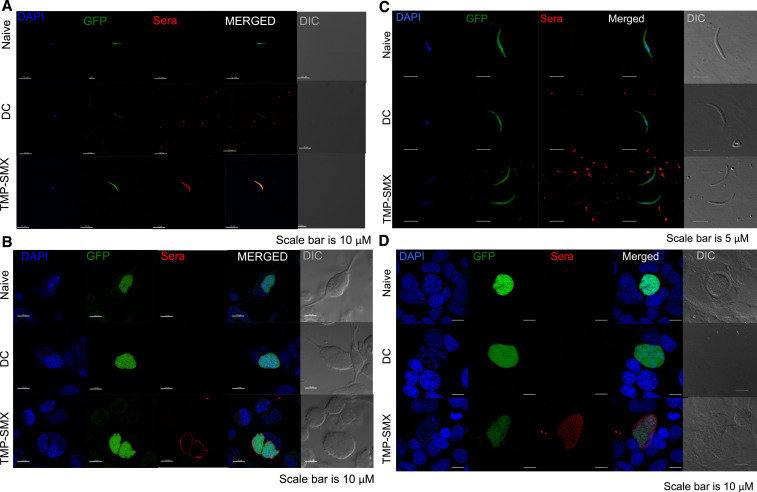
Antibody generated by TMP-SMX-CVac recognizes antigens from sporozoites (SPZ) and liver stages (exoerytrocytic forms, EEFs) of homologous parasites, but only EEFs of heterologous parasites. GFP-expressing *P. yoelii* (**A**: SPZ and **B**: EEFs) and *P. berghei* (**C**: SPZ and **D**: EEFs) SPZ and EEFs were immunostained with either serum (at a dilution of 1:400) from control/nave or TMP-SMX-CVac immunized mice and antibody recognition was detected using Alexa-594 conjugated anti-mouse IgG (red signal). EEF images were acquired 48h post-SPZ infection of either HepG2 (*P. berghei*) or HepG2:CD81 (*P. yoelii*). DAPI was used to stain the nucleus of both parasite and host cells. Chemoprophylaxis vaccination (CVac)/trimethoprim–sulfamethoxazole (TMP-SMX) mouse sera recognizes *Plasmodium yoelii* SPZs and exoerythrocytic forms EEFs (**A** and **B**), but only *Plasmodium berghei* EEFs (**C** and **D**). DC: Drug control; Trimethoprim-Sulfamethoxazole-CVac immunized; DIC = differential interference contrast image.

To examine whether CVac sera recognized antigens of homologous and heterologous species, we then conducted IFA experiments. Immunofluorescence assay suggested that CVac immune sera recognized antigens from both with *P. yoelii* SPZs and EEFs (Figure 2A and B). Antibody staining of *P. yoelii* EEFs (2B) appeared to be sharply limited inside of the hepatocyte, whereas that of controls (naive or DC) did not. Chemoprophylaxis vaccination/TMP-SMX immune sera did not recognize *P. berghei* SPZs, but there was again evidence of some recognition of EEFs, although with more diffuse staining across the EEF (Figure 2C and D).

## CONCLUSIONS/DISCUSSION

Drug-based attenuation of malaria parasites and resultant protective immunity has been demonstrated in animals and humans, but the mechanisms remain unclear. Using the *P. yoelii* CVac/TMP-SMX model, we show that mouse immune sera have partial SPZ neutralization activity.

Although CVac sera did not confer sterilizing immunity, it partially reduced the development of homologous EEFs in vitro and in vivo. However, CVac sera failed to confer protective activity against heterologous species infection in vitro or in vivo. These data could suggest that insufficient antibody was present to prevent patency.

The lack of heterologous protection could also be due to significant interspecies heterogeneity between the antigens to which antibody responses were directed.

In the irradiated SPZ immunization model, mouse immune sera recognized only homologous SPZs and did not mediate protection against heterologous challenge.^16^ Indeed, our IFAs indicate that reactogenicity differs between species with the use of CVac serum, indicating that there was enough difference between species-specific proteins in EEFs and SPZs. However, how that translates into functional immune responses cannot be directly extrapolated, especially because the IFAs were fixed.

Prior work, including our own, however, also suggests protective heterologous responses in human *Plasmodium falciparum* CVac trials and mainly due to cell-not humoral-mediated immune responses.^17^ In line with this, in both this CVac/TMP-SMX model, and the irradiated SPZ model, CD8 T cells make an important contribution to sterile immunity.^7^ Interestingly, our prior publications using this CVac model clearly indicate that cell-mediated responses are at play in protecting against heterologous and homologous challenges, and that an antibody response was not sufficient to elicit a protective response.^7^

Notably, both our CVac model and irradiated SPZ model contrast with other models of CVac systems in which the blood-stage antimalarial chloroquine is used. In that case, antibody mediates parasite neutralization in vivo and protection and against heterologous challenge.^18^ However, for the latter CVac model, blood-stage mediated protection likely plays a larger role because cross-stage immunity is observed in this model in contrast to our CVac/TMP-SMX model.^7,18^ Overall, therefore, the potential contribution of CD8 T- or CD4 T-cell direct lytic activity, or CD4 T-cell–mediated help, for antibody production, as shown in rodent models,^19^ cannot be excluded, and likely depends on the model used.

In the field, TMP-SMX prophylaxis confers protection against malaria to varying degrees in areas of different transmission intensities, even where the prevalence of antifolate resistance mutations is high.^2,3^ However, the contribution of the developing immune response to malaria protection remains unclear. Dissecting the immune response in mice receiving repeated SPZ inoculation while on TMP-SMX prophylaxis has its value; such animal model studies are better poised to identify protective responses.^20^ However, clinical studies are essential to determine the impact and extent of TMP-SMX effect on antimalarial immunity development. Indeed, future clinical studies are needed to understand whether and how TMP-SMX prophylaxis modifies the development of an antimalarial immune response in malaria-endemic areas.
